# Gold nanoparticles stabilized with βcyclodextrin-2-amino-4-(4-chlorophenyl)thiazole complex: A novel system for drug transport

**DOI:** 10.1371/journal.pone.0185652

**Published:** 2017-10-11

**Authors:** I. Asela, M. Noyong, U. Simon, J. Andrades-Lagos, J. Campanini-Salinas, D. Vásquez-Velásquez, M. Kogan, N. Yutronic, R. Sierpe

**Affiliations:** 1 Departamento de Química, Facultad de Ciencias, Universidad de Chile, Santiago, Chile; 2 Institute of Inorganic Chemistry, RWTH Aachen University, Aachen, Germany; 3 Departamento de Química Farmacológica y Toxicológica, Facultad de Ciencias Químicas y Farmacéuticas, Universidad de Chile, Santiago, Chile; 4 Advanced Center for Chronic Diseases (ACCDiS), Universidad de Chile and Pontificia Universidad Católica de Chile, Santiago, Chile; Institute of Materials Science, GERMANY

## Abstract

While 2-amino-4-(4-chlorophenyl)thiazole (AT) drug and thiazole derivatives have several biological applications, these compounds present some drawbacks, such as low aqueous solubility and instability. A new complex of βCD-AT has been synthesized to increase AT solubility and has been used as a substrate for the deposit of solid-state AuNPs via magnetron sputtering, thus forming the βCD-AT-AuNPs ternary system, which is stable in solution. Complex formation has been confirmed through powder X-ray diffraction and 1D and 2D nuclear magnetic resonance. Importantly, the amine and sulfide groups of AT remained exposed and can interact with the surfaces of the AuNPs. The complex association constant (970 M^-1^) has been determined using phase solubility analysis. AuNPs formation (32 nm average diameter) has been studied by UV-Visible spectroscopy, transmission/scanning electron microscopy and energy-dispersive X-ray analysis. The *in vitro* permeability assays show that effective permeability of AT increased using βCD. In contrast, the ternary system did not have the capacity to diffuse through the membrane. Nevertheless, the antibacterial assays have demonstrated that AT is transferred from βCD-AT-AuNPs, being available to exert its antibacterial activity. In conclusion, this novel βCD-AT-AuNPs ternary system is a promising alternative to improve the delivery of AT drugs in therapy.

## Introduction

The concept of drug delivery is not only linked to the investigation of entry and release processes, but also to the creation of increasingly complex and efficient mechanisms that support the entire drug-administration cycle—from the generation of a pharmaceutical productto elimination from the organism. Optimizing drug administration requires, for example, that novel delivery systems and technologies be able to regulate the rate-of-release and site-specific targeting of a drug. Optimized regulation would achieve more effective therapeutic treatments with fewer adverse effects as a consequence of minimized dosing frequencies/quantities and increased drug concentrations at the site-of-action [1–2].

**Fig 1:**
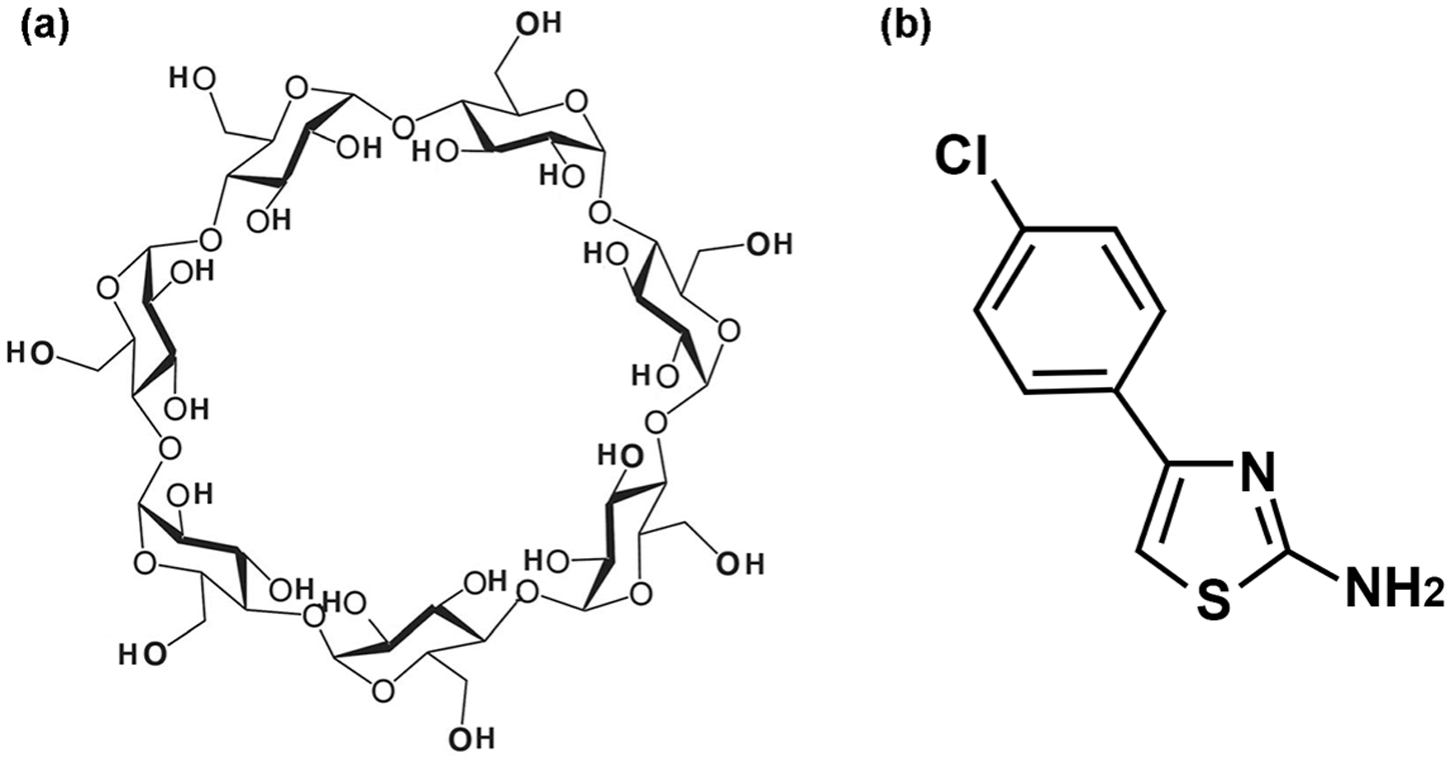
Structural representation of (A) βCD and (B) AT.

Different strategies are being investigated to prevent or minimize undesirable drug properties. One such approach is the inclusion of drugs into cyclodextrins, macromolecules that can form inclusion complexes (ICs) modifying the physical, chemical, and/or biological properties of the included drug [3–6]. The most commonly used is the βcyclodextrin (βCD), which has applications in pharmaceuticals, food stuffs, and environmental protection, among many others [7–10]. Specifically, βCD is a water soluble, nontoxic cyclic oligosaccharide conformed of 7 glucopyranose units (Fig 1A) observable as a three-dimensional bucket-like shape, with primary hydroxyl groups in the narrowest extreme and secondary hydroxyls groups in the widest extreme. The βCD cavity, the inner walls of which are partially hydrophobic, possesses CH and glycosidic oxygen groups [11]. Furthermore, βCD can include diverse non polar molecules of appropriate dimensions that can increase in solubility in water by forming ICs. These guest molecules may also be protected against oxidation, photolysis, or enzymatic degradation, among other processes. Altogether, these properties broaden its possible biological applications [12–17].

Ongoing pharmaceutical research has reported constantly on the formation of βCD complexes and its possible advantages/applications [18]. The drugs that act as guests must have suitable dimensions for inclusion in βCD, that is, the region of the drug included should have a smaller dimension than the matrix cavity (between 6 and 6.5 Å approximately), among them, molecules with a structural thiazole group. The wide variety of thiazole structures exists due to the high reactivity and ease-of-synthesis of these compounds [19–20]. Antifungal, antibacterial, and anti-inflammatory biological functions, as well as hypertension, cancer, and HIV treatment potentials, have been studied for this compound family [19, 21–22]. One thiazole derivative is the drug 2-amino-4-(4-chlorophenyl)thiazole (AT) (Fig 1B). This compound showed antibacterial activity in different strains in a qualitative analysis using disk diffusion method [23]. Nevertheless, AT showed no activity in a quantitative analysis using broth microdilution method [24], probably due to their low solubility. In this sense, the use of cyclodextrins allows increasing aqueous solubility, and even improving photostability, bioavailability of drugs and eventually to reduce side effects [25]. The inclusion of antibiotics in βCD potentiates the antibacterial activity through various mechanisms [26–28]. Notably, the AT molecule is an investigative model for other derivatives and, additionally, has suitable functional groups that may interact with metal nanoparticles to form nanometric systems with potential biomedical applications.

Indeed, nanoparticles represent an interesting alternative for changing the physicochemical properties of some materials or compounds, thereby forming a system with applications in drug delivery [29]. Among the different nanostructures subject to ongoing investigation, gold nanoparticles (AuNPs) stand out. AuNPs can be synthesized with different sizes and shapes and, due to well-control able surface chemical reactivity, can be functionalized with various ligands, including with polymers, drugs, or genetic material, to develop multifunctional therapeutic strategies [30–34]. When exposed to light, AuNPs react to the electromagnetic field by a collective oscillation of electrons, a phenomenon termed surface plasmon resonance. In turn, surface plasmon resonance causes a strong absorption of the incident light, and a portion of this energy is dissipated as local heat, along with the release of molecules from the AuNP surface. Due to these properties, AuNPs are applied in nanomedicine for detection, using techniques such as surface-enhanced Raman spectroscopy [35] or surface-enhanced fluorescence [36]; as plasmonic sensors [37]; for drug transport and release [38–40]; or in photothermal treatments [41–43].

Successful biomedical applications of AuNPs require understanding the mechanisms of uptake in AuNP-based systems and the dependence of said systems on variables such as size, surface charge, and molecules/compound bound on the AuNPs’ surface. In general, the cellular uptake of AuNPs is inversely proportional to the size of this nanoparticle, where systems sized between 10 to 50 nm are more effective for drug transport and cellular internalization [44–45]. However, it is important to mention size effectiveness strongly depends on the type of cell line studied. Therefore, properly designed AuNP systems can interfere in biological processes at the nanoscale level, which would lead to treatment and diagnostic advances for multiple diseases. In this sense, AuNPs have been used to improve the pharmacokinetic and pharmacodynamic properties of antibiotics in several publications [46–49], since allow increased drug concentration at infected sites as well as reduce toxicity of the antibiotic. One stabilization method for AuNPs is through the use of supramolecular cyclodextrin systems, an approach that has been studied recently for different purposes, such as the inclusion of drugs [50–53].

Considering the presented context, it is proposed the construction of a drug carrier based on βCD and AuNPs, using the AT molecule as a model. Novel βCD-AT complex formation has been characterized using x-ray diffraction and 1D/2D nuclear magnetic resonance (NMR). It has been postulated that the functional groups of the guest included in the matrix would stabilize AuNPs produced by sputtering, thereby forming the βCD-AT-AuNPs ternary system. Subsequently, the AuNPs have been dissolved to form a colloidal solution stabilized by the ICs. This ternary system has been constructed with suitable conditions for potential biomedical applications (e.g. nanometric size, drug-cyclodextrin association, etc.). Additionally, the βCD-AT-AuNPs system may serve as a nanocarrier for different drugs. Specifically regarding AT, incorporation into the βCD-AT-AuNPs system may result in increased water solubility, stability, and effective permeability in artificial membranes. As a proof of concept, the system has been studied in different bacteria, it is expected that the drug will be released demonstrating antibacterial activity even in bacteria where pure AT has been inactive.

## Materials and methods

### Inclusion complex formation

The IC has been synthesized with βCD hydrated (98% purity, 1134.98 g/mol) and AT (98%purity, 216.68 g/mol), both obtained from Sigma-Aldrich. The used solvents are analytical-grade ethanol and Milli-Q water, both obtained from Merck. The IC has been prepared in a saturated solution that mixed the matrix and guest in a 1:1 molar ratio. βCD (0.600 g) has been dissolved in water with constant stirring at room temperature. In turn, AT (0.114 g) has been dissolved in ethanol with constant stirring at room temperature. Both solutions have been mixed and maintained under fume hood for two weeks until producing a crystalline powder. This crystalline powder has been filtered by adding ethanol to remove any excess AT or free βCD. Finally, the formed IC has been dried under a vacuum and stored in an amber vial. The obtained fine-powder crystals have been characterized by powder x-ray diffraction (PXRD) and 1D/2D NMR.

### Gold nanoparticles formation using sputtering

**Fig 2:**
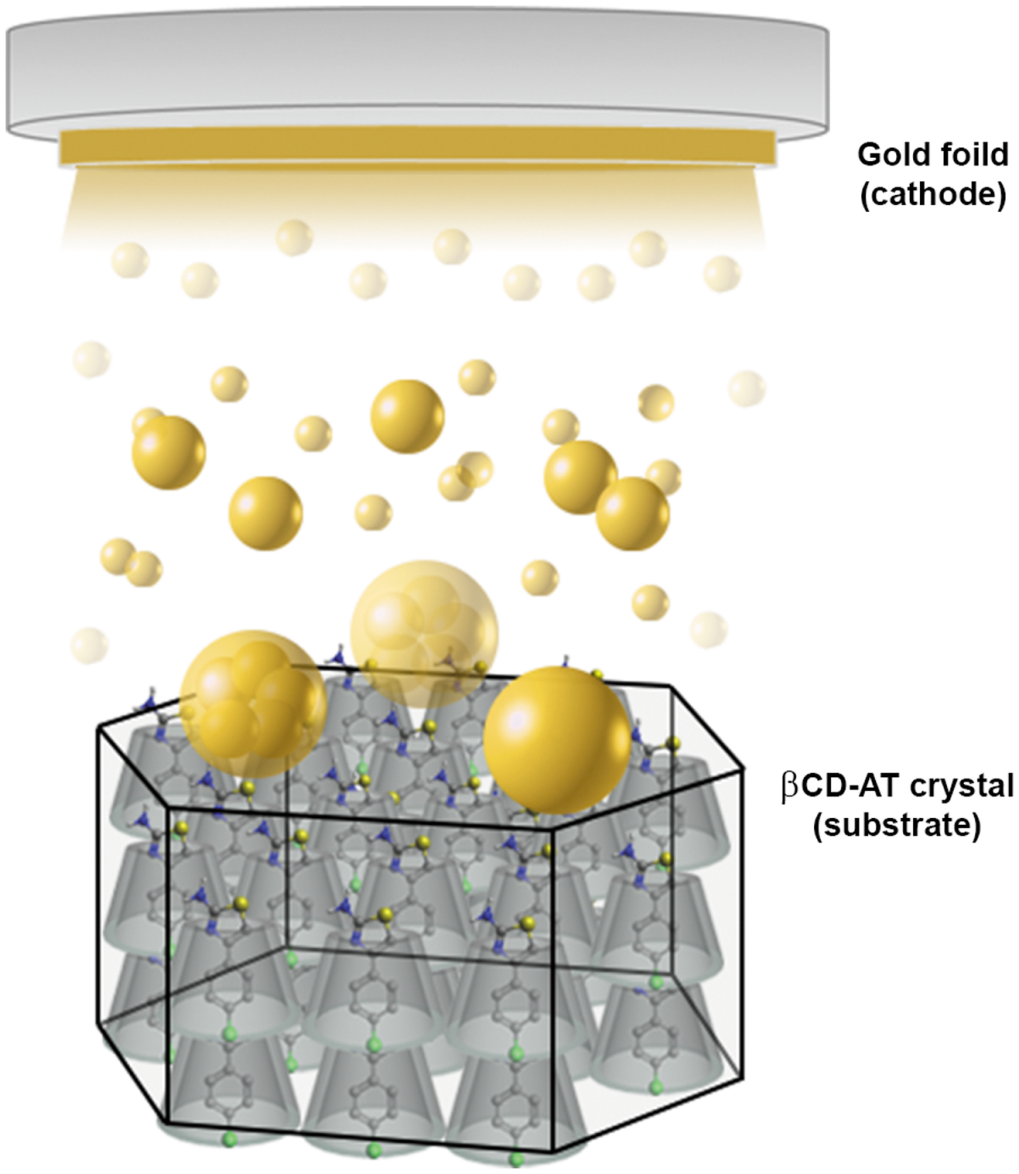
βCD-AT-AuNPs ternary system. Schematic representation of AuNPs deposited via sputtering on crystalline powder of βCD-AT forming the ternary system.

The AuNPs have been obtained via magnetron sputtering in high vacuum using a Pelco SC6sputter coater (Cressington Scientific Instruments) with a gold foil (99.99% purity). Approximately 10 mg of the complex have been pulverized in an agate mortar, and applied as a crystalline powder to thinly layer a glass slide in the sputter coater. AuNPs have been deposited onto βCD-ATs using a gold foil as a cathode and complex-derived crystalline powder as a substrate. Both the foil and substrate have been placed inside the vacuum chamber at 0.5 mbar. A 25 mA current generated an ionized argon atmosphere, thus initiating the sputtering process. Over 30 s, with 5 s intervals, AuNPs have been deposited onto the crystalline powder substrate, resulting in the formation of the βCD-AT-AuNPs ternary system (Fig 2), with all crystal faces covered by nanoparticles. The obtained system has been characterized by UV-Visible, PXRD, transmission and scanning electron microscopy (TEM and SEM, respectively), and energy-dispersive x-ray analysis.

### Powder x-ray diffraction

Powder x-ray diffractograms have been acquired in a Siemens D5000 diffractometer with graphite-monochromated Cu Kα radiation at 40 kV and 30 mA, with a wavelength of 1.540598 Å.

### Nuclear magnetic resonance

^1^H-NMR and rotating-frame Overhauser spectroscopy (ROESY) measurements have been acquired at 300 K in dimethyl sulfoxide-d_6_ (99.99% D) on a Bruker ADVANCE 400 MHz superconducting NMR spectrometer. 2D NMR spectra have been obtained using pulsed field gradient-selected methods during a mixing time of 12 hours. Furthermore, complex stoichiometry has been evaluated through the integration of βCD and AT proton signals in the βCD-AT spectrum.

### UV-Visible spectroscopy in the solid state

Samples with AuNPs on βCD-AT crystals have been characterized on a UV spectrophotometer (model UV-2450, Shimadzu Scientific Instruments) via diffuse reflectance, using barium sulfate as a baseline. Absorbance has been determined based on Kubelka-Munk transformation with the UVProbe Software (Shimadzu Scientific Instruments).

### Scanning electron microscopy and energy dispersive x-ray

SEM micrographs have been obtained using a LEO 1420VP system equipped with an Oxford 7424 energy dispersive spectrometer at an accelerating voltage of 25 kV.

### Field emission scanning electron microscopy

Field emission SEM (FE-SEM) micrographs have been obtained in a Leo Zeiss SUPRA35VP microscope at acceleration voltages of 15kV and 2kV.

### Transmission electron microscopy

TEM micrographs have been obtained using a JEOL JEM 1200 EX II microscope. This instrument operates at low pressures (i.e. 10^−4^ to 10^−8^kPa). All samples have been measured at an acceleration voltage of 80 kV. For preparation, the samples (≈ 1.0 mg) have been dispersed in isopropanol (1.0 mL, 20%). Then, a drop (10 μL) has been deposited onto a copper grid with a continuous parlodon film; any excess solution has been removed, and the grid has been dried. Studies on size distribution have been also performed (counting approximately 200 nanoparticles).

### Parallel artificial membrane permeability assays

Transwell plates have been used with donor and acceptor wells, the latter of which had a semipermeable polyvinylidene fluoride membrane. Exactly 4 μL of a phosphatidylcholine in dodecane (20 mg/mL) solution has been deposited onto the polyvinylidene fluoride membranes of the acceptor plate. The plate has been kept still for approximately five minutes until the complete evaporation of dodecane, after which, PBS (300 μL, pH = 7.4) has been added. Each PBS-dissolved sample (300 μL) has been deposited in the donor well. The studied samples have been the AT drug, the βCD-AT complex, and the βCD-AT-AuNPs ternary system. A thiopental solution (10 mM; 10 mg in 100 mL PBS) has been used as a positive control, while an Evans blue solution (10 mM; 200 mg in 100 mL PBS) served as a negative control. The donor and acceptor wells have been loaded (samples/controls and PBS, respectively), assembled, covered, and sealed using parafilm. Each parallel artificial membrane permeability assay (in triplicate, n = 3) has been performed over 24 h at 37°C with constant agitation at 280 rpm.

Effective permeability (*P*_*e*_) has been determined using Eq (1):

$${\text{P}}_{\text{e}}=\frac{-218.3}{\text{t}}\;\cdot \;\text{log}\left[1-\frac{2\;\cdot \;{\text{C}}_{\text{A}}\left(\text{t}\right)}{{\text{C}}_{\text{D}}\left(0\right)}\right]\;\cdot \;10^{-6}\text{cm}/\text{s}$$ (1)

Where *t* is the time in hours, *C*_*A*_ is the concentration in the acceptor plate at *t*, and C_D_ is the concentration in the donor plate at 0 *t*. Concentrations in the acceptor plate have been calculated by measuring the absorbance of the different plates using a UV-Visible spectrophotometer and by the respective calibration curve of AT.

### Bacterial strains

For the screening of antibacterial activity, the following strains have been used: methicillin-resistant *Staphylococcus aureus* ATCC 43300, methicillin-sensitive *Staphylococcus aureus* (MRSA) ATCC 29213, *Enterococcus faecalis* ATCC 29212, *Eschericha coli* ATCC 25922, *Pseudomonas aeruginosa* ATCC 27853 and *Klebsiella pneumoniae* ATCC 700603.

### Antibacterial activity determination

The Minimum Inhibitory Concentrations (MIC) has been determined using a broth microdilution method, according to Clinical and Laboratory Standards Institute (CLSI) [54]. Vancomycin and gentamycin have been used as quality control, according to MIC ranges reported by CLSI [55]. All compounds tested have been dissolved in DMSO-water, not exceeding 2% for well. The inoculum has been prepared to a turbidity equivalent of 0.5 McFarland standard, diluted in broth media to give a final concentration of 5×10^5^ CFU/mL in the test tray, then covered and placed in plastic bags to prevent evaporation. The plates have been incubated at 35°C for 18–20 h. The MIC has been defined as the lowest concentration of compound giving complete inhibition of visible growth. All the experiments were performed three times (in triplicate).

## Results and discussion

### Formation of theβCD-AT complex in the solid state

**Fig 3:**
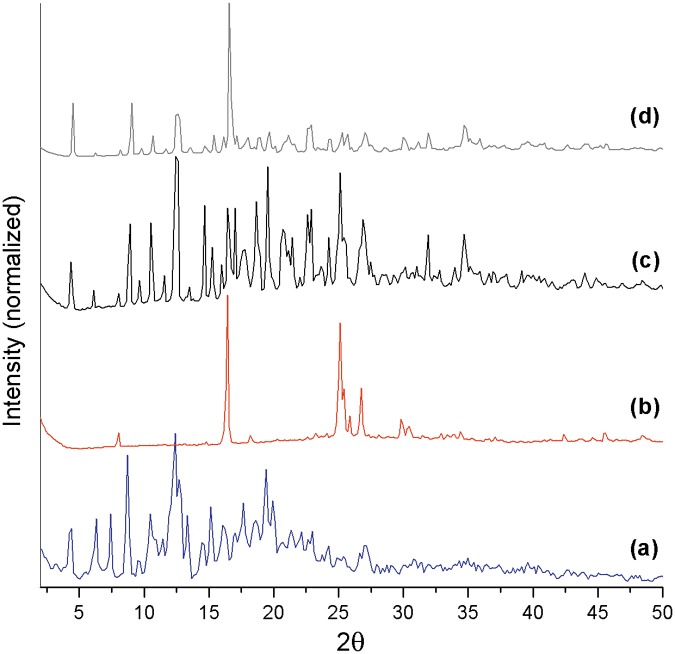
Powder x-ray diffraction (PXRD) intensities. PXRD diffractograms of (A) βCD, (B) AT,(C) the βCD-AT complex, and (D) the physical βCD/AT mixture.

To demonstrate the formation of an IC using PXRD, a comparison between different diffraction patterns is necessary. During comparison, observable differences should exist between the traces of the matrix, guest, and supposed complex, such as the disappearance of the characteristic peaks of pure compounds or appearance of new reflexes. Such analysis is based on the fact that the crystalline packing of the IC is different from the original compounds [56]. In the present study, diffractograms have been obtained for βCD, AT, the βCD-AT complex, and the physical βCD and AT mixture (Fig 3A–3D, respectively). Differences have been observed between the traces of the pure species (Fig 3A and 3B) and the IC (Fig 3C) due to the inclusion of AT in the interior of the matrix cavity, which formed a new crystal structure. AT (Fig 3B) had a highly crystalline order, with an intense peak at approximately17° 2θ. The trace obtained for the physical mixture (Fig 3D) superimposed this intense peak against the diffraction pattern of βCD and it is clearly different from the trace of the formed complex (Fig 3C).The crystallographic pattern of the βCD-AT complex (Fig 3C) has been indexed for a monoclinic type P2_1_ system [57], the network parameters of which are specified in the S1 Appendix.

### Study of the βCD-AT complex in solution

**Fig 4:**
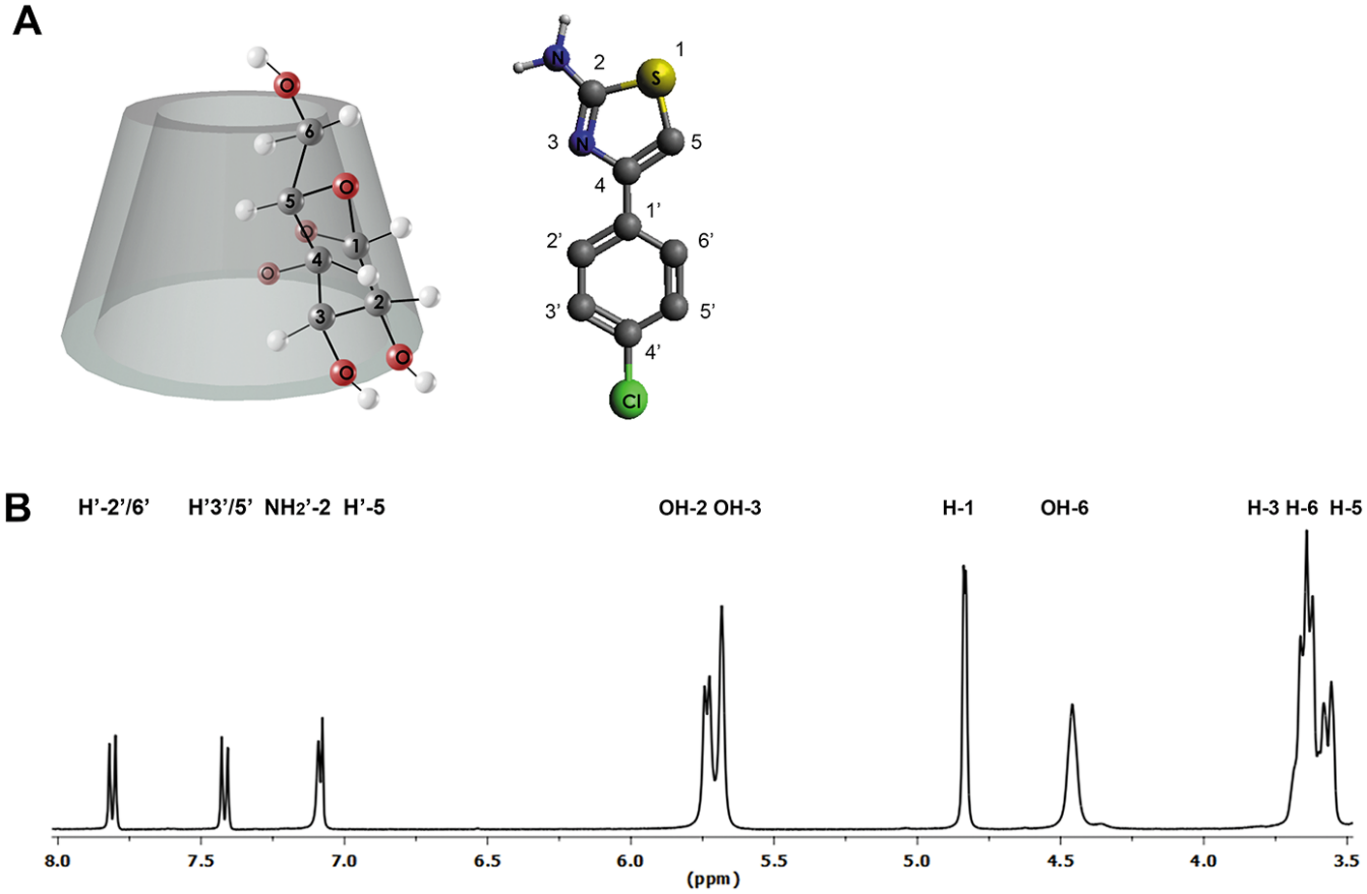
(A) Schematic representation of βCD and AT protons, and (B) ^1^H-NMR spectrum of the βCD-AT complex.

After demonstrating complex formation in the solid state, the crystalline powder of βCD-AT has been solubilized and analyzed by NMR to confirm that the complex permanence in solution. The proton skeletons of AT and βCD with one glucopyranose unit are shown in Fig 4A.

Fig 4B shows the ^1^H-NMR spectrum of the βCD-AT complex in solution. A complete displacement analysis has been possible for all of the protons involved in the inclusion process, specifically of the protons of AT and the H-3, H-5 and H-6 protons of βCD which are inwardly oriented and the hydroxyl groups which are found in the exteriors of the cavity. The chemical shifts of the IC and free species are compared (Table 1). The signals corresponding to the H-3 and H-5 protons of βCD shifted to lower fields, whereas all hydroxyl group signals and the H-6 proton signal of the matrix appeared at higher fields due to interaction with the drug. The spectra of the pure species are shown in Fig A in S2 Appendix.

**Table 1:** Chemical shifts of the IC and free species.

H of βCD	δ βCD (ppm)	δ βCD-AT (ppm)	Δδ (ppm)	H’ of AT	δ AT (ppm)	δ βCD-AT (ppm)	Δδ (ppm)
**H-3**	3.648	3.655	0.007	**H´-5**	7.065	7.058	-0.007
**H-5**	3.555	3.561	0.006	**H’-2'/6'**	7.802	7.797	-0.005
**H-6**	3.617	3.612	-0.005	**H’-3'5'**	7.410	7.405	-0.005
**OH-2**	5.735	5.726	-0.009	**NH**_**2**_**’-2**	7.065	7.066	0.001
**OH-3**	5.680	5.675	-0.005				
**OH-6**	4.479	4.451	-0.028				

Additionally, the 1:1 stoichiometry of the complex has been calculated by integrating the ^1^H-NMR spectrum signals. Specifically, the integration of the H-1 proton of the matrix has been used as a reference and compared with integration results for protons of the AT aromatic ring (S3 Appendix).

The association constant of the IC has been determined using the phase solubility method, as described by Higuchi and Connors [58]. The value of the constant K has been 970 M^−1^, which proves to be suitable for potential applications in drug delivery (S4 Appendix). Matrix-guest association constants must be between 50 and 2000 M^-1^ for cyclodextrins to have potential pharmaceutical uses. For example, if the formed complex is unstable (at lower values, <50 M^−1^), the drug could be released early, or, if the association is very high (with values >2000 M^−1^), the cavity could retain the drug, thus impeding release at the site-of-action. In either case, controlled drug release does not occur [59–60].

**Fig 5:**
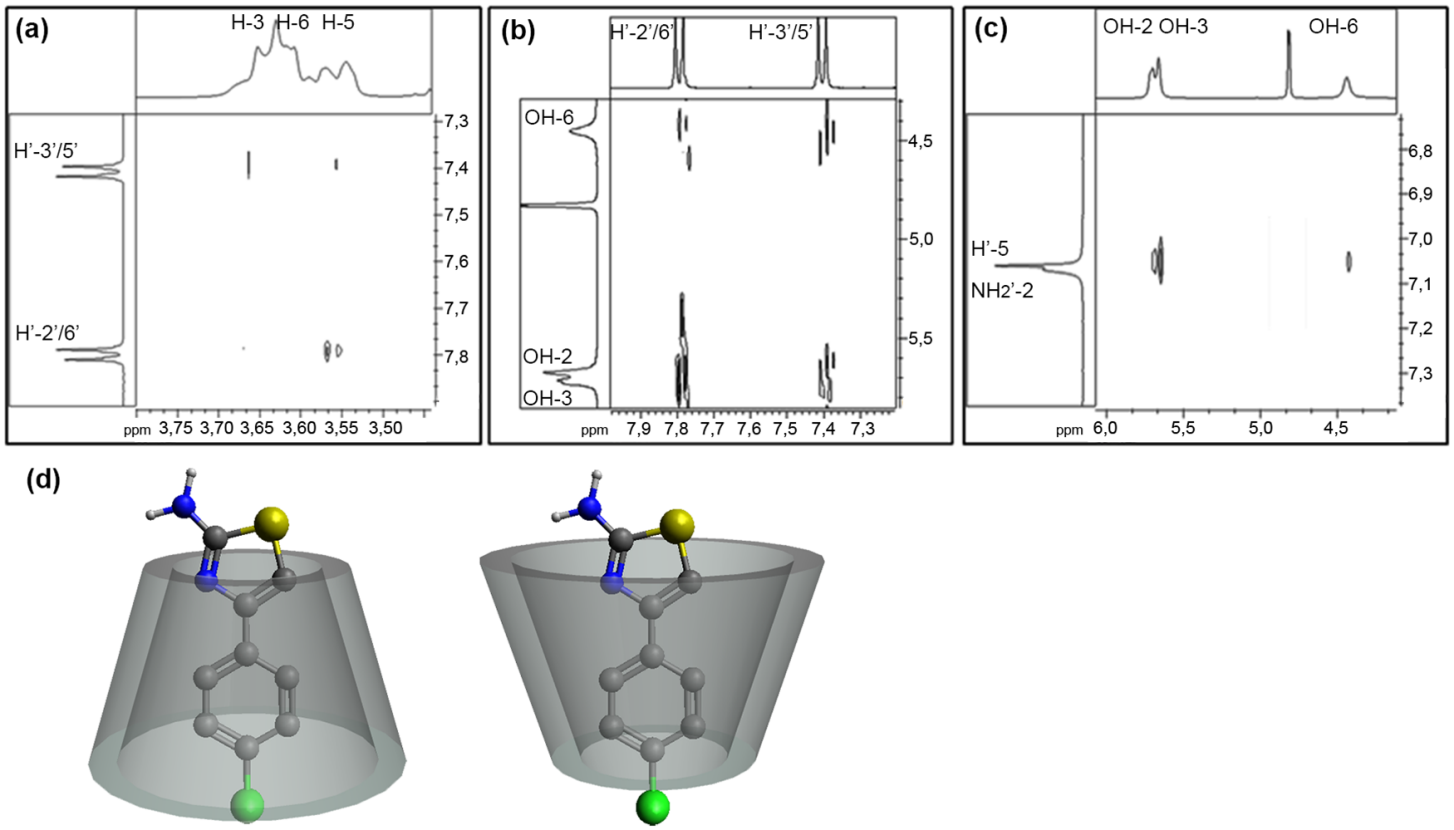
ROESY spectra of the βCD-AT complex and proposed inclusion geometries. Cross peaks between: (A) the H-3, H-5 and H-6 protons of βCD with the H’-2’/3’/5’/6’ protons of the aromatic ring of AT, (B) OH-2 and OH-3 groups of βCD with the H’-2’/3’/5’/6’ protons of the aromatic ring of AT, (C) OH-2 and OH-3 groups of βCD with NH_2_’-2 and H'-5 protons of the thiazole group of AT.(D) Proposed inclusion geometries of the βCD-AT complex.

ROESY analysis reveals interactions between the hydrogen nuclei of structures that are not chemically bonded and are located at distances not greater than 5 Å. Therefore, this technique can be used to determine the geometric structure of ICs in solution with a high degree of precision [61]. Fig 5 shows the cross peaks of the βCD protons, as well as the H’-2’/3’/5’/6’ and NH_2_’-2 protons of AT forming the IC. More specifically, Fig 5A shows the cross peaks produced by the interaction between the inner protons of βCD and the H'-2'/ 6' and H'-3'/ 5' protons comprising the aromatic ring of AT, thus demonstrating the effective inclusion of the drug. In turn, the spectrum in Fig 5B shows the correlation between the aromatic ring of AT and the primary/secondary hydroxyl groups of βCD, which implies that the drug is included near the narrow and wide cavities of the bucket. Finally, Fig 5C shows the cross peaks between the H'-5 proton of AT and all hydroxyl group protons of the matrix. An intense correlation between the OH-2 and OH-3 groups of βCD and the H'-5 proton of AT has been observed. A less intense correlation has been also found between the OH-6 groups and H'-5 proton, the full ROESY spectrum is shown in Fig B in S2 Appendix.

**Fig 6:**
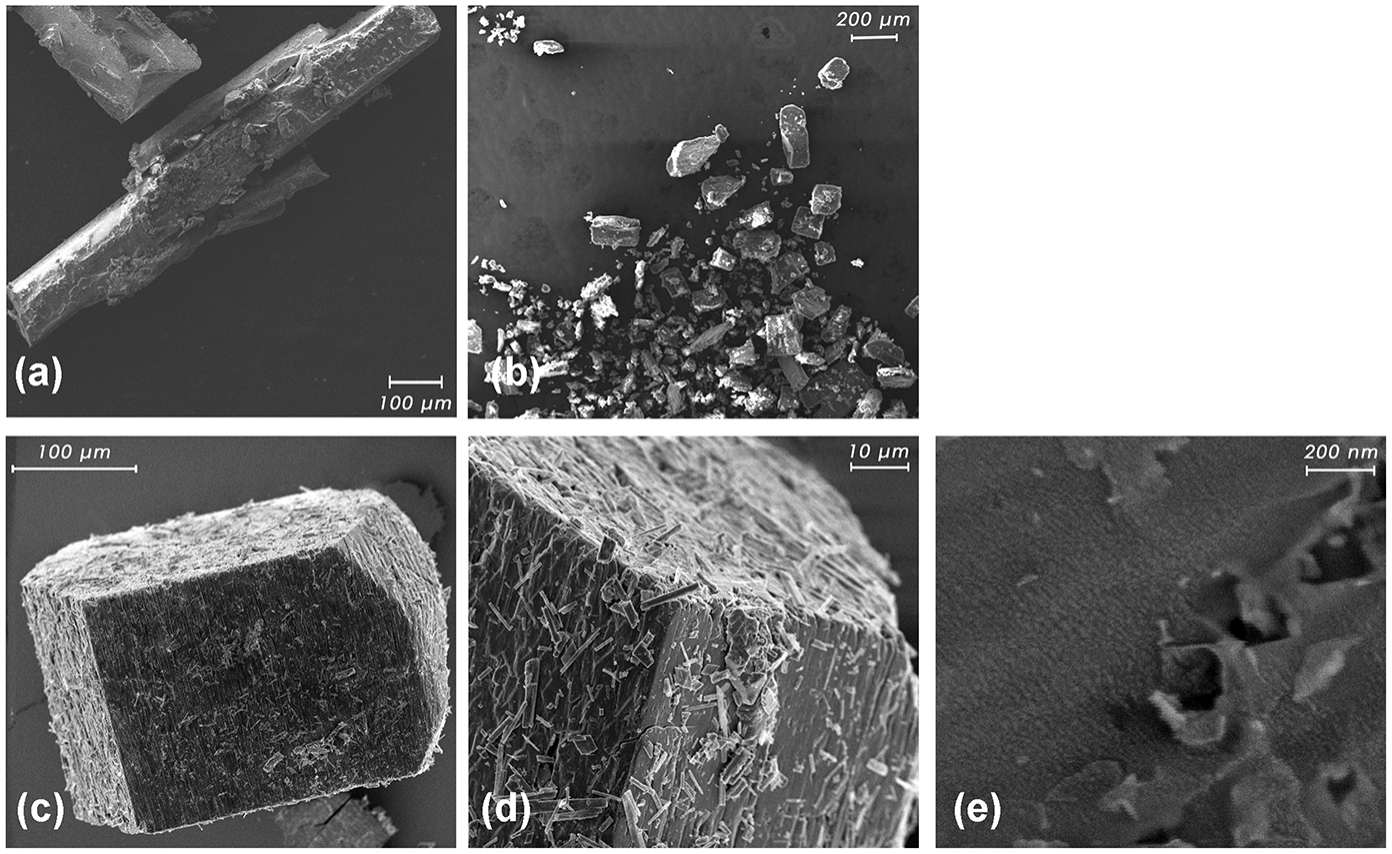
SEM micrographs. Shown are (A) pure AT, (B) pure βCD, (C, D), βCD-AT crystals, and (E) AuNPs deposited on the βCD-AT crystals.

Based on analysis of the obtained ROESY spectra and of the chemical shifts evidenced by ^1^H-NMR, two possible inclusion structures have been proposed (Fig 6D).

### Deposit of AuNPs onβCD-AT complex crystals

AuNPs have been deposited through the physical sputtering method over different periods of time (5 to 30 s, in 5 s intervals). Various times have been assessed to establish the optimum time length in which the deposited particles would be nanometric in size and with a proportional relation between the width of the plasmon and absorption intensity observed in the UV-Visible spectrum. All UV-Visible spectra obtained at different sputtering times are shown in S5 Appendix.

Assessments of the βCD-AT-AuNPs ternary system in the solid state through SEM and FE-SEM images are shown in Fig 6. Contrasting with the morphology of the pure AT and βCD species (Fig 6A and 6B), the complex with AuNPs presented the characteristic morphology of these crystals (Fig 6C and 6D). These observations are consistent with the obtained PXRD results, which show a different diffraction pattern in each pure species and in the formed complex (see Fig 3). Using high-resolution images, AuNPs deposited on βCD-AT crystals can be directly observed (Fig 6E).

**Fig 7:**
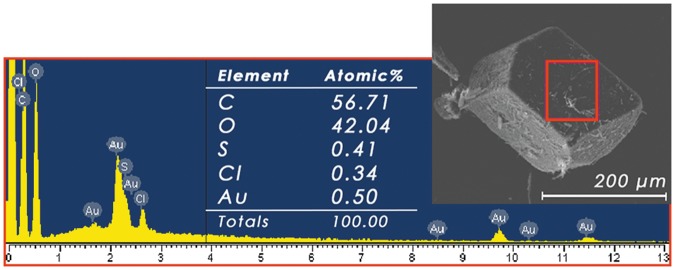
Energy-dispersive x-ray analysis. Analysis (left) of the crystal (boxed area) for the IC with AuNPs. No further signals have been detected for energies lager than 13 keV.

Energy-dispersive x-ray analysis has been performed on different faces of the crystals to deduce the percentages of each component in the sample (Fig 7). AuNPs deposited with an optimal sputtering time (30 s), Au and all of the elements constituting the IC have been observed.

### Study of the βCD-AT-AuNPs ternary system

**Fig 8:**
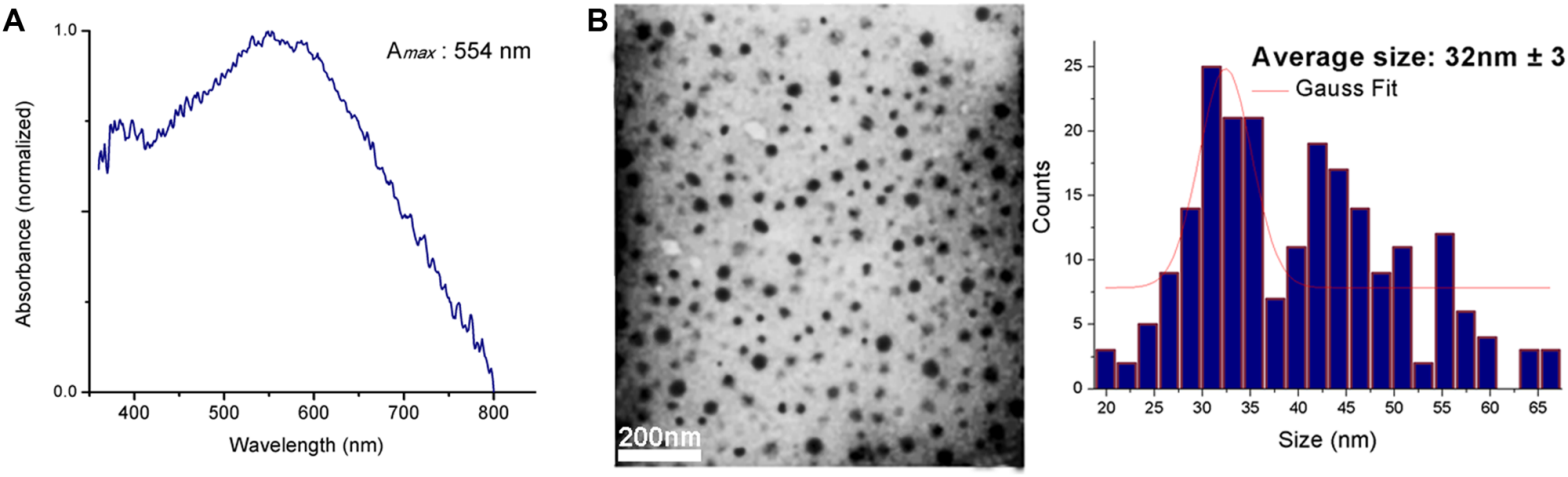
AuNPs absorbance spectrum and TEM data. (A) Absorbance spectrum of AuNPs deposited on IC microcrystals using sputtering for 30 s. (B)TEM micrograph (left) of AuNPs covered with the IC (20000X magnification) and the respective histogram (right).

Fig 8A shows the absorbance spectrum of AuNPs deposited onto βCD-AT complex microcrystals over a sputtering time of 30 s, with maximum absorbance at 554 nm (S5 Appendix). The position of the absorbance band may be due to the change in the surrounding medium, even when AuNPs are supported on the ICs, or also due to the phenomenon of interparticle coupling caused by the increased proximity between nanoparticles in the solid state [62–64], which has been directly observed using FE-SEM.

The ternary system has been dissolved, and the AuNPs form a colloidal solution that is stabilized by the ICs. Fig 8B shows the TEM micrographs of AuNPs with an average diameter size of 32 nm (±3 nm) and low polydispersity. Additionally, TEM imaging revealed that the nanoparticles are not aggregated and remained stable in solution when covered by the IC.

Gold spherical nanoparticles measuring10 nm in diameter exhibits a maximum UV-Visible absorbance at 520 nm [65]. The bathochromic displacement of the absorbance of AuNPs covered with the complex would be due to an increased particle size, as observed by TEM. In addition to this, while half-band width is mainly attributed to high polydispersity, the current observations indicate that half-band width is due to interparticle coupling produced by the proximity of the AuNPs deposited on the solid complex.

**Fig 9:**
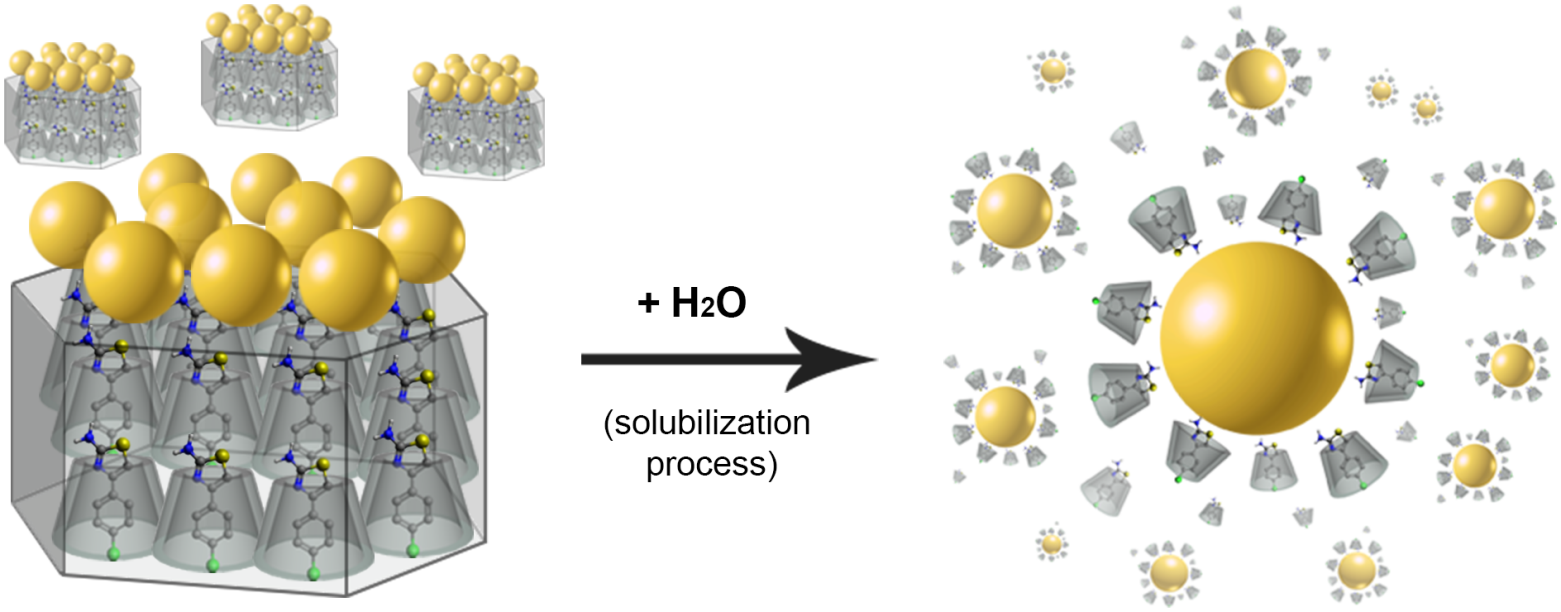
Solubilization process of the AuNPs. Schematic representation of solubilized AuNPs covered with βCD-AT complexes from crystals of the ICs with AuNPs deposited on its surface.

Considering that the AuNPs have been initially deposited on crystals of the IC, without another precursor, the subsequent solubilization of this system allows the particles to be covered only by the complexes [52, 66], remaining stable, with form and size discussed above by TEM. A scheme that represents the colloidal solution obtained from AuNPs in the solid state is shown in Fig 9.

**Fig 10:**
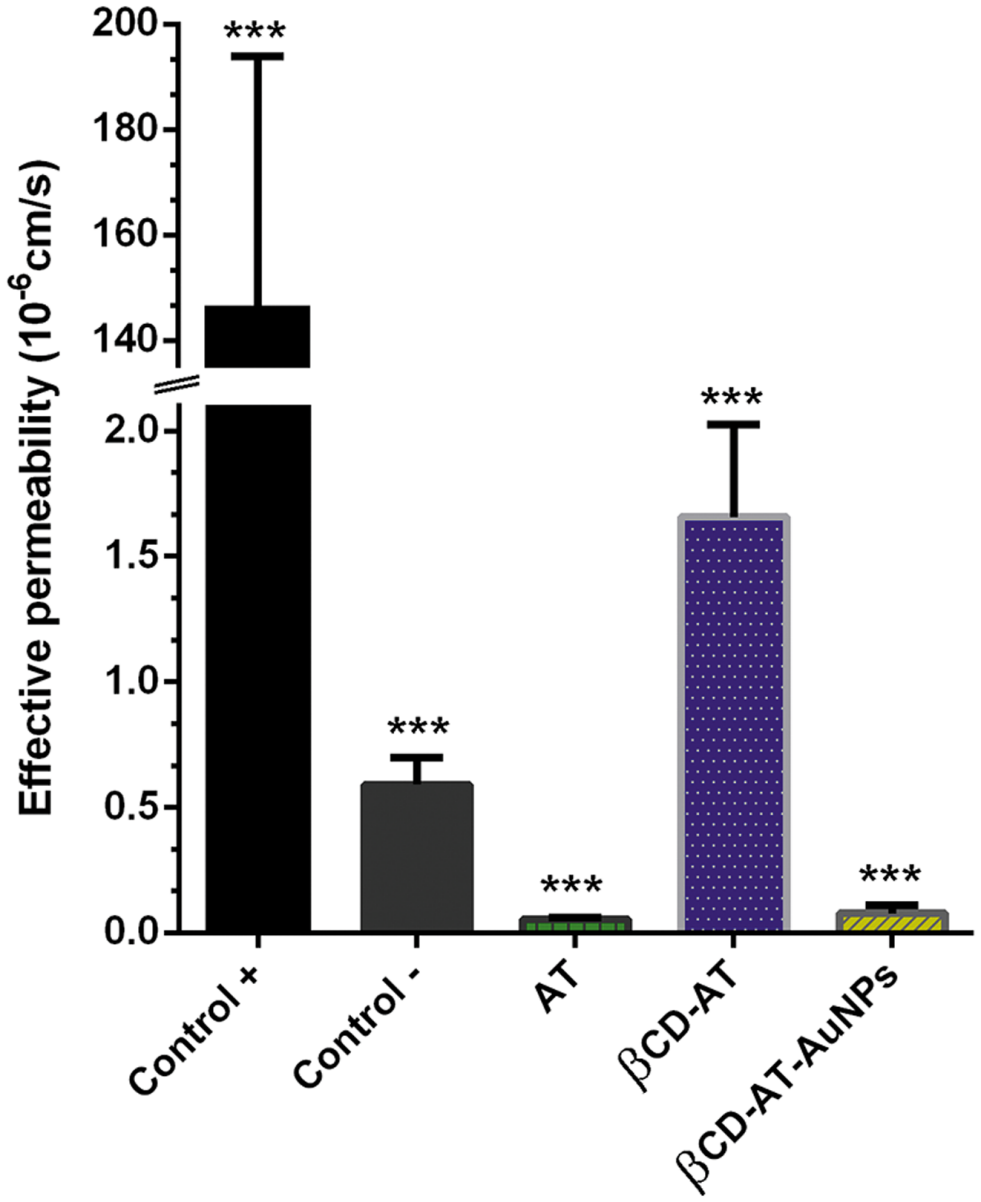
Effective permeability measurements. Shown are the effective permeability for the AT drug, βCD-AT complex, and βCD-AT-AuNPs system, as well as the corresponding positive (+) and negative (-) controls.

To evaluate permeability in artificial membranes, a parallel artificial membrane permeability assay has been performed for free AT, with βCD, and with AuNPs. For this assay, a phosphatidylcholine lipid membrane has been constructed to simulate the lipid bilayer of different cell types. Since the artificial membrane in the plate contain no active transport system or metabolic enzymes, it is only possible to study the passive diffusion of species [67–68]. It has been reported that cyclodextrins can improve drug permeation through biological membranes under certain conditions; this depends, for example, of the type of cyclodextrin, association constant, unstirred water layer on the phospholipid surface or stirring rate [69–70]. The obtained results demonstrated that the low effective permeability of AT increased when included in βCD (Fig 10), which is relevant to improve the cell penetration of the drug. The βCD-AT-AuNPs ternary system showed low effective permeability, equivalent to the free drug. This has been an expected result since AuNPs of the assessed sizes do not passively diffuse through lipid membranes [51], which corroborates the possibility of constructing nanoparticulate systems that promote site-specific drug arrival, as controlled by other transport types (e.g. active diffusion). All absorbances and concentrations obtained can be found in the S6 Appendix.

In order to evaluate whether AT maintains its antibacterial activity *in vitro*, after the functionalization with cyclodextrins and gold nanoparticles, the broth microdilution test has been performed to determine the minimum inhibitory concentration (MIC) of each compound in 6 bacteria of clinical relevance. The results of antibacterial activity for tested compounds are listed in Table 2.

**Table 2:** Antibacterial activity the βCD-AT-AuNPs, βCD-AT and AT.

Compound	MIC (μg/mL)					
	SA^a^	MRSA^b^	EF^c^	EC^d^	PA^e^	KP^f^
**AT**	›64	›64	64	›64	›64	›64
**βCD-AT**	1024	1024	64	›1024	›1024	1024
**βCD-AT-AuNPs**	1024	1024	64	1024	1024	1024
**vancomycin**	0.5	0.5	0.5	-	-	-
**gentamicin**	-	-	-	0.5	0.25	0.5

^a^methicillin-sensitive *Staphylococcus aureus* ATCC 29213,

^b^methicillin-resistant *Staphylococcus aureus* ATCC 43300,

^c^*Enterococcus faecalis* ATCC 29212,

^d^*Eschericha coli* ATCC 25922,

^e^*Pseudomonas aeruginosa* ATCC 27853, and

^f^ *Klebsiella pneumoniae* ATCC 700603.

The highest concentration tested has been 64 μg/mL for AT and 1024 μg/mL for βCD-AT-AuNPs and βCD-AT. The ranges of MIC values observed were 1024 to 64 μg/ml. The results show that AT only exhibited activity on *E*. *faecalis* (MIC = 64 μg/ml). When AT is complexed with βCD, forming βCD-AT, antibacterial activity (MIC = 64 μg/ml) is maintained, this shows that compound AT is released from βCD, becoming available to exert its activity. Similarly, when βCD-AT is functionalized with AuNPs, forming βCD-AT-AuNPs also maintains the antibacterial activity, demonstrating that the presence of AuNPs does not intervene in the antibacterial activity of the drug. AT had no activity in SA, MRSA, EC, PA or KP at the concentrations tested, the result being limited due to the low solubility of the compound in the test medium. This result agrees with what has been previously reported in the literature [24].

The obtainment of βCD-AT improves solubility 16 times of AT (in 2% DMSO/H_2_O), even reaching concentrations of 1024 μg/ml. This allows to reach higher concentrations in the assay, observing antibacterial activity in most of the strains tested. The βCD-AT-AuNPs system showed activity in all strains tested at 1024 μg/ml. The concentrations of all solutions prepared can be found in the S7 Appendix.

## Conclusions

Solid-state complex formation between the drug 2-amino-4-(4-chlorophenyl)thiazole (AT) and βcyclodextrin (βCD) has been demonstrated by powder x-ray diffraction. Nuclear magnetic resonance verified that the complex remained stable in solution, with an association constant of 970 M^-1^.This is an optimal value for applications in drug delivery, where βCD can be used to enhance drug solubility, stability, safety, and bioavailability. Two possible dispositions of AT inside of the matrix have been shown by inclusion geometry obtained via rotating-frame Overhauser spectroscopy. In both cases, the functional groups of AT have been exposed, thus allowing for gold nanoparticles (AuNPs) formation and deposition, as obtained by sputtering in a high vacuum. Our study also supports the subsequent stabilization of the AuNPs by inclusion complexes. In the solid state, AuNPs have been deposited on the surface faces of βCD-AT crystals, which are then dissolved. The complex-coated AuNPs remained stable and presented an average size of 32 nm. Furthermore, the βCD-AT-AuNPs ternary system had dimensions suitable for potential biomedical applications.

We argue that nanoparticulate systems can be used for the transport and release of different drugs, with respect to AT, increasing its stability and aqueous solubility within organisms, among other therapeutic advantages. Specifically, the parallel artificial membrane permeability assays demonstrated that the effective permeability of AT can be increased by βCD. In turn, the incorporation of AuNPs prevented the permeation of the ternary system through passive diffusion. The study of antibacterial activity shows that AT can be transferred in an in vitro system, allowing to exert its biological effect since its value of MIC in *Enterococcus faecalis* remained constant after the functionalization. In addition, using βCD-AT-AuNPs the solubility of AT has been improved 16-fold in the assay medium, obtaining antibacterial activity at 1024 ug/mL in all evaluated bacteria.

## Supporting information

S1 Appendix Powder x-ray diffraction. The diffractogram of βCD-AT has been indexed in a monoclinic type P2_1_ system using the Powder X program, to refine the system onto a theoretical network with parameters corresponding to the βCD-sulfathiazole complex (βCD-ST), which belongs to the same isostructural family series [56]. The theoretical and experimental parameters are specified in Table A. After network parameter refinement, 16 intense peaks have been found between 2° and 50° for 2θ (Fig A).The respective details for hkl assignment, angles, distances, and relative intensities are detailed in Table B.
(PDF)

S2 Appendix Nuclear magnetic resonance spectroscopy. The ^1^H-NMR spectra of the complex and pure species, in DMSO-d_6_, are shown in Fig A. Full 2D ROESY spectrum of βCD-AT (Fig B) confirmed the formation of the IC and has been used to determine the exact arrangement of the guest inside the βCD cavity.
(PDF)

S3 Appendix βCD-AT complex stoichiometry. The 1:1 molar ratio has been calculated by integrating ^1^H-NMR spectrum signals of the βCD-AT complex, using the H-1 proton of βCD as a reference for comparison with protons of the AT aromatic ring. Fig A shows that the H-1 proton of the matrix integrates for 7, as per the 7 comprising glucose units. In turn, CH groups of the aromatic ring of the guest integrate for 2 as these groups are pairs of equivalent protons.
(PDF)

S4 Appendix βCD-ATcomplex association constant. Phase solubility method has been used to obtain the association constant of βCD-AT, which has a 1:1 molar stoichiometry. First, the molar extinction coefficient (ε_AT_) of AT has been calculated. Fig A (left) shows the different known concentrations of AT in an aqueous solution. From the slope of the line, ε_AT_ has been obtained (21.91 ±0.7 mmol^-1^∙L∙cm^-1^). Subsequently, eight supersaturated solutions of AT have been prepared. Different βCD concentrations have been added to each solution to increase the aqueous solubility of the drug. The AT/βCD mixtures have been stirred constantly for 24 hours and then allowed to rest for an additional hour. The aqueous phase containing the drug solubilized in water and the drug included in βCD have been separated and analyzed by UV-Visible spectroscopy. Maximum absorbance (at 234 nm), the ε_AT_, and the Lambert-Beer equation are used to obtain the AT concentrations for all assays. Finally, the differing concentrations of βCD and AT included in each complex have been plotted (Fig A right). The value of the slope in the graph for βCD/AT has been 0.0512 (SE 0.003). The association constant has been calculated considering reported mathematical analysis [58] and using the slope value obtained in the graph. The value for the K_1:1_ of the βCD-AT complex has been 970 M^-1^.
(PDF)

S5 Appendix UV-Visible spectroscopy in the solid state. AuNPs formation on βCD-AT microcrystals has been studied via UV-Visible in the solid state (Fig A). The different exposure times of samples to sputtering have been registered by diffuse reflectance (350 to 800 nm wavelength). Then, absorbance has been obtained through Kubelka-Munk transformation.
(PDF)

S6 Appendix Parallel artificial membrane permeability assay. PAMPA has been performed in triplicate (n = 3), with each n representing an average of three assays performed on the same plate. Table A shows the absorbance values, calculated concentrations and effective permeabilities of the different evaluated systems.
(PDF)

S7 Appendix Antibacterial activity the βCD-AT-AuNPs,βCD-AT and AT. For the preparation of the solutions of AT, βCD-AT and βCD-AT-AuNPs, these compounds have been weighed according to the mass ratios as shown in Table A. The concentrations of all solutions prepared have been made in function of mass of AT. Fig A shows that the functionalization with βCD and AuNPs does not affect the antibacterial activity of AT (MIC = 64 μg/ml). This indicates that the compound can be released from the system and exert its activity against *Enterococcus faecalis*.
(PDF)
