# Supplementary material for: Gold nanoparticles stabilized with βcyclodextrin-2-amino-4-(4-chlorophenyl)thiazole complex: A novel system for drug transport
Source: PLoS One. 2017 Oct 11;12(10):e0185652. doi: 10.1371/journal.pone.0185652 (PMC5636091; doi:10.1371/journal.pone.0185652)
Supplement: S1 Appendix — The diffractogram of βCD-AT has been indexed in a monoclinic type P21 system using the Powder X program, to refine the system onto a theoretical network with parameters corresponding to the βCD-sulfathiazole complex (βCD-ST), which belongs to the same isostructural family series [56]. The theoretical and experimental parameters are specified in Table A. After network parameter refinement, 16 intense peaks have been found between 2° and 50° for 2θ (Fig A).The respective details for hkl assignment, angles, distances, and relative intensities are detailed in Table B. (PDF) [file pone.0185652.s001.pdf]

S1 Appendix. Powder x-ray diffraction

The diffractogram of βCD-AT has been indexed in a monoclinic type $P2_1$system using the Podwer X program, to refine the system onto a theoretical network with parameters corresponding to the βCD-sulfathiazole complex (βCD-ST), which belongs to the same isostructural family series [45]. The theoretical and experimental parameters are specified in Table A. After network parameter refinement, 16 intense peaks have been found between 2° and 50° for 2θ (Fig A). The respective details for hkl assignment, angles, distances, and relative intensities are detailed in Table B.

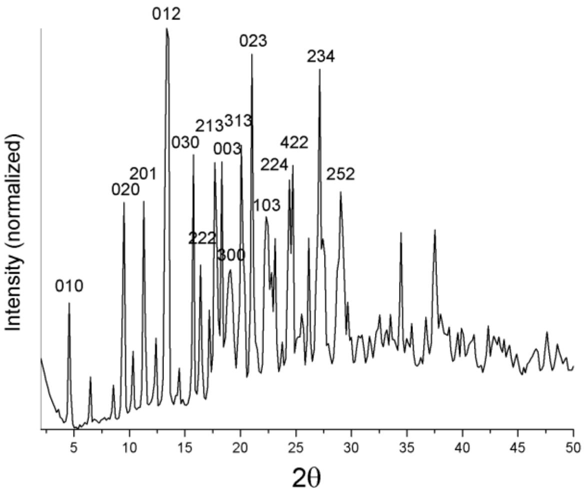

Fig A. Indexed diffractogram of the βCD-AT complex.

Table A. Theoretical and experimental network parameters of the studied systems.

Network parameters	α=γ	β	a(Å)	b(Å)	c(Å)	Volume (Å^3)	R factor
Theoretical of βCD	90°	112.22°	21.233	10.294	15.103	3301.1	---
Theoretical of βCD-ST	90°	117.29°	15.264	16.500	15.559	3918.6	---
Experimental of βCD-AT	90°	117.29°	15.303	16.509	15.504	3916.9	0.0041

Table B. hkl planes, angles, and intensities of βCD-AT.

hkl	Experimental angle (2θ)	Calculated angle (2θ)	Difference (2θ)	Experimental distance (Å)	Calculated distance (Å)	Intensity percentage (norm.)
010	4.884	5.349	-0.465	18.079	16.509	55.5
020	9.912	10.709	-0.797	8.916	8.255	48.6
201	11.820	11.565	0.255	7.481	7.646	70.0
012	13.866	13.918	-0.052	6.382	6.358	100.0
030	16.398	16.093	0.304	5.402	5.503	47.4
300	17.126	17.226	-0.100	5.173	5.144	30.7
222	18.478	18.456	0.022	4.798	4.803	88.5
213	19.103	19.311	-0.208	4.642	4.593	47.2
003	19.657	19.567	0.091	4.513	4.533	31.5
313	20.941	20.948	-0.007	4.239	4.237	64.8
023	21.877	22.132	-0.255	4.059	4.013	65.8
103	23.160	23.089	0.071	3.837	3.849	46.8
224	25.345	25.397	-0.052	3.511	3.504	39.8
422	25.657	25.660	-0.003	3.469	3.469	50.3
234	28.154	28.166	-0.012	3.167	3.166	57.9
252	30.235	30.265	-0.030	2.954	2.951	38.4
