# Supplementary material for: Gold nanoparticles stabilized with βcyclodextrin-2-amino-4-(4-chlorophenyl)thiazole complex: A novel system for drug transport
Source: PLoS One. 2017 Oct 11;12(10):e0185652. doi: 10.1371/journal.pone.0185652 (PMC5636091; doi:10.1371/journal.pone.0185652)
Supplement: S2 Appendix — The 1H-NMR spectra of the complex and pure species, in DMSO-d6, are shown in Fig A. Full 2D ROESY spectrum of βCD-AT (Fig B) confirmed the formation of the IC and has been used to determine the exact arrangement of the guest inside the βCD cavity. (PDF) [file pone.0185652.s002.pdf]

S2 Appendix. Nuclear magnetic resonance spectroscopy

The ^{1}H-NMR spectra of the complex and pure species, in DMSO-d_6, are shown in Fig A.

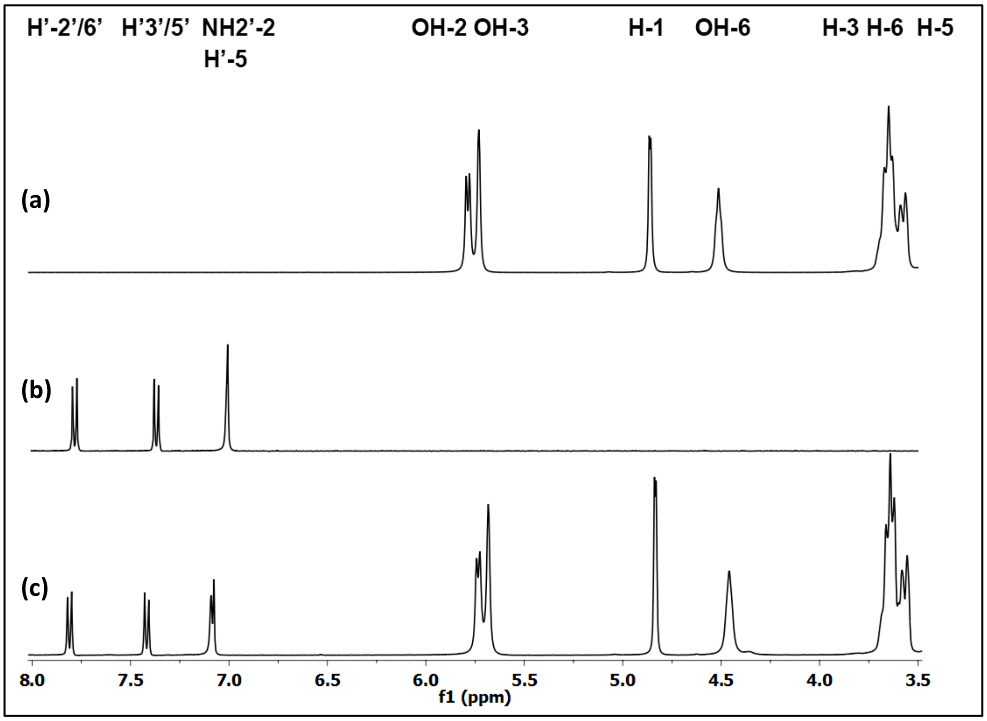

Fig A. ^{1}H-NMR spectra of (a) pure βCD, (b) pure AT, and (c) βCD-AT in DMSO-d_6.

Full 2D ROESY spectrum of βCD-AT (Fig B) confirmed the formation of the IC and has been used to determine the exact arrangement of the guest inside the βCD cavity.

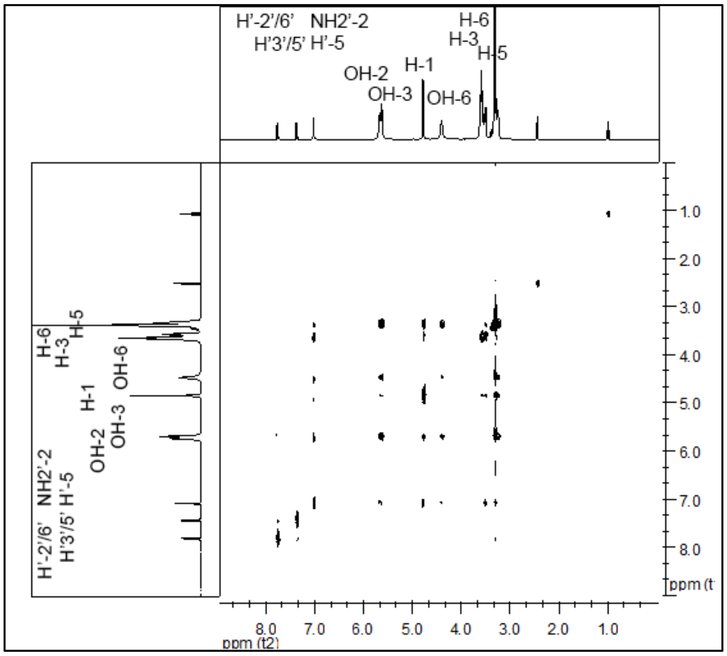

Fig B. Full ROESY spectrum of the βCD-AT complex in DMSO-d₆.
