# Supplementary material for: Gold nanoparticles stabilized with βcyclodextrin-2-amino-4-(4-chlorophenyl)thiazole complex: A novel system for drug transport
Source: PLoS One. 2017 Oct 11;12(10):e0185652. doi: 10.1371/journal.pone.0185652 (PMC5636091; doi:10.1371/journal.pone.0185652)
Supplement: S3 Appendix — The 1:1 molar ratio has been calculated by integrating 1H-NMR spectrum signals of the βCD-AT complex, using the H-1 proton of βCD as a reference for comparison with protons of the AT aromatic ring. Fig A shows that the H-1 proton of the matrix integrates for 7, as per the 7 comprising glucose units. In turn, CH groups of the aromatic ring of the guest integrate for 2 as these groups are pairs of equivalent protons. (PDF) [file pone.0185652.s003.pdf]

S3 Appendix. βCD-AT complex stoichiometry

The 1:1 molar ratio has been calculated by integrating ^{1}H-NMR spectrum signals of the βCD-AT complex, using the H-1 proton of βCD as a reference for comparison with protons of the AT aromatic ring. Fig A shows that the H-1 proton of the matrix integrates for 7, as per the 7 comprising glucose units. In turn, CH groups of the aromatic ring of the guest integrate for 2 as these groups are pairs of equivalent protons.

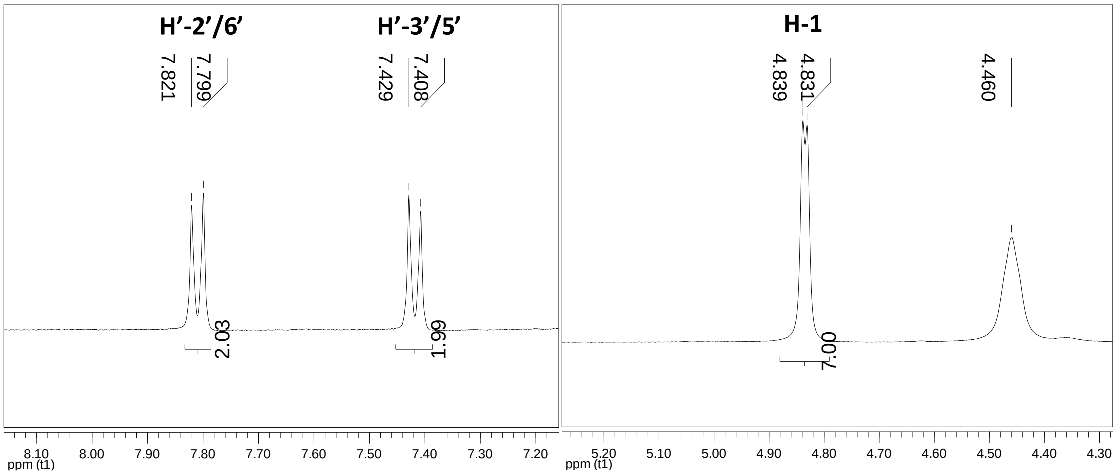

Fig A. Integration of the ^{1}H-NMR spectrum of βCD-AT in DMSO-d_6.
