# Supplementary material for: Gold nanoparticles stabilized with βcyclodextrin-2-amino-4-(4-chlorophenyl)thiazole complex: A novel system for drug transport
Source: PLoS One. 2017 Oct 11;12(10):e0185652. doi: 10.1371/journal.pone.0185652 (PMC5636091; doi:10.1371/journal.pone.0185652)
Supplement: S4 Appendix — Phase solubility method has been used to obtain the association constant of βCD-AT, which has a 1:1 molar stoichiometry. First, the molar extinction coefficient (εAT) of AT has been calculated. Fig A (left) shows the different known concentrations of AT in an aqueous solution. From the slope of the line, εAT has been obtained (21.91 ±0.7 mmol-1∙L∙cm-1). Subsequently, eight supersaturated solutions of AT have been prepared. Different βCD concentrations have been added to each solution to increase the aqueous solubility of the drug. The AT/βCD mixtures have been stirred constantly for 24 hours and then allowed to rest for an additional hour. The aqueous phase containing the drug solubilized in water and the drug included in βCD have been separated and analyzed by UV-Visible spectroscopy. Maximum absorbance (at 234 nm), the εAT, and the Lambert-Beer equation are used to obtain the AT concentrations for all assays. Finally, the differing concentrations of βCD and AT included in each complex have been plotted (Fig A right). The value of the slope in the graph for βCD/AT has been 0.0512 (SE 0.003). The association constant has been calculated considering reported mathematical analysis [58] and using the slope value obtained in the graph. The value for the K1:1 of the βCD-AT complex has been 970 M-1. (PDF) [file pone.0185652.s004.pdf]

S4 Appendix. βCD-AT complex association constant

Phase solubility method has been used to obtain the association constant of βCD-AT, which has a 1:1 molar stoichiometry. First, the molar extinction coefficient (ε_{AT}) of AT has been calculated. Figure A (left) shows the different known concentrations of AT in an aqueous solution. From the slope of the line, ε_{AT} has been obtained (21.91 ±0.7 $mmol^{-1}\cdot L\cdot cm^{-1}$).

Subsequently, eight supersaturated solutions of AT have been prepared. Different βCD concentrations have been added to each solution to increase the aqueous solubility of the drug. The AT/βCD mixtures have been stirred constantly for 24 hours and then allowed to rest for an additional hour. The aqueous phase containing the drug solubilized in water and the drug included in βCD have been separated and analyzed by UV-Visible spectroscopy. Maximum absorbance (at 234 nm), the ε_{AT}, and the Lambert-Beer equation are used to obtain the AT concentrations for all assays. Finally, the differing concentrations of βCD and AT included in each complex have been plotted (Fig A right). The value of the slope in the graph for βCD/AT has been 0.0512 (±0.003). The association constant has been calculated considering reported mathematical analysis [47] and using the slope value obtained in the graph. The value for the $K_{1:1}$ of the βCD-AT complex has been 970 M^{-1}.

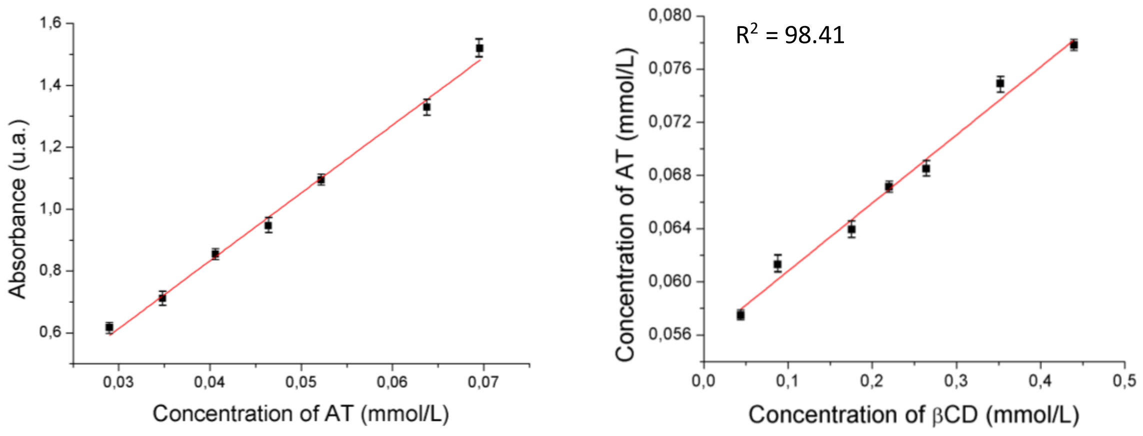

Fig A. Linear plot following the phase solubility method. (Left) maximum absorbance levels for AT at 324 nm versus concentrations of AT; and (right) different concentrations of AT versus concentration of βCD.
