# Supplementary material for: Gold nanoparticles stabilized with βcyclodextrin-2-amino-4-(4-chlorophenyl)thiazole complex: A novel system for drug transport
Source: PLoS One. 2017 Oct 11;12(10):e0185652. doi: 10.1371/journal.pone.0185652 (PMC5636091; doi:10.1371/journal.pone.0185652)
Supplement: S5 Appendix — AuNPs formation on βCD-AT microcrystals has been studied via UV-Visible in the solid state (Fig A). The different exposure times of samples to sputtering have been registered by diffuse reflectance (350 to 800 nm wavelength). Then, absorbance has been obtained through Kubelka-Munk transformation. (PDF) [file pone.0185652.s005.pdf]

## S5 Appendix. UV-Visible spectroscopy in the solid state

AuNPs formation on βCD-AT microcrystals has been studied via UV-Visible in the solid state (Fig A). The different exposure times of samples to sputtering have been registered by diffuse reflectance (350 to 800 nm wavelength). Then, absorbance has been obtained through Kubelka-Munk transformation.

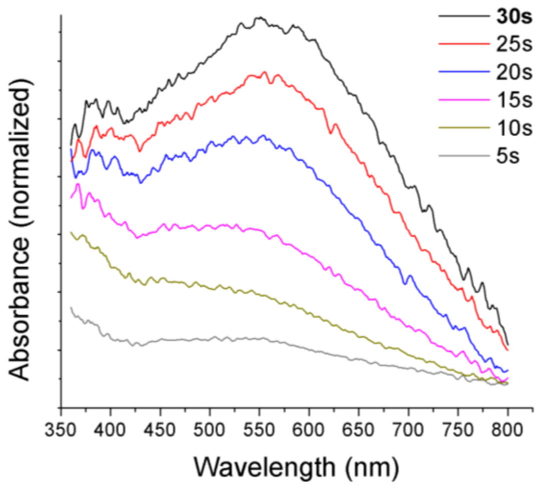


**Fig A. Absorbance spectra of AuNPs on βCD-AT for different sputtering times.**
