# Supplementary material for: Gold nanoparticles stabilized with βcyclodextrin-2-amino-4-(4-chlorophenyl)thiazole complex: A novel system for drug transport
Source: PLoS One. 2017 Oct 11;12(10):e0185652. doi: 10.1371/journal.pone.0185652 (PMC5636091; doi:10.1371/journal.pone.0185652)
Supplement: S6 Appendix — PAMPA has been performed in triplicate (n = 3), with each n representing an average of three assays performed on the same plate. Table A shows the absorbance values, calculated concentrations and effective permeabilities of the different evaluated systems. (PDF) [file pone.0185652.s006.pdf]

S6 Appendix. Parallel artificial membrane permeability assay

PAMPA has been performed in triplicate (n = 3), with each n representing an average of three assays performed on the same plate. Table A shows the absorbance values, calculated concentrations and effective permeabilities of the different evaluated systems.

Table A. Absorbances, concentrations and effective permeabilities obtained via parallel artificial membrane permeability assay

System	Absorbance (u.a.)	Concentration (µmol/mL)	Effective permeability (cm/s)
AT	0.0936	0.0128	0.0644×10^{-6}
	0.0663	0.0091	0.0456×10^{-6}
	0.0705	0.0096	0.0485×10^{-6}
βCD-AT	0.2211	0.0303	2.067×10^{-6}
	0.1760	0.0241	1.549×10^{-6}
	0.1572	0.0215	1.351×10^{-6}
βCD-AT-AuNPs	0.0277	0.0019	0.1018×10^{-6}
	0.0114	0.0008	0.0416×10^{-6}
	0.0240	0.0016	0.0887×10^{-6}
