# Supplementary material for: Gold nanoparticles stabilized with βcyclodextrin-2-amino-4-(4-chlorophenyl)thiazole complex: A novel system for drug transport
Source: PLoS One. 2017 Oct 11;12(10):e0185652. doi: 10.1371/journal.pone.0185652 (PMC5636091; doi:10.1371/journal.pone.0185652)
Supplement: S7 Appendix — For the preparation of the solutions of AT, βCD-AT and βCD-AT-AuNPs, these compounds have been weighed according to the mass ratios as shown in Table A. The concentrations of all solutions prepared have been made in function of mass of AT. Fig A shows that the functionalization with βCD and AuNPs does not affect the antibacterial activity of AT (MIC = 64 μg/ml). This indicates that the compound can be released from the system and exert its activity against Enterococcus faecalis. (PDF) [file pone.0185652.s007.pdf]

S7 Appendix. Antibacterial activity the βCD-AT-AuNPs, βCD-AT and AT

For the preparation of the solutions of AT, βCD-AT and βCD-AT-AuNPs, these compounds have been weighed according to the mass ratios as shown in Table A. The concentrations of all solutions prepared have been made in function of mass of AT.

Table A. Mass ratio of AT, βCD-AT and βCD-AT-AuNPs.

Compound	Mass Ratio
AT	mAT[a]
βCD-AT	mAT = 0,158 · mβCD-AT[b]
βCD-AT-AuNPs	mAT = 0,158 · (0,993 · mβCD-AT-AuNPs[c])

[a]AT mass, [b]βCD-AT mass; considering a 1: 1 molar ratio of the complex where 84.2% m/m is βCD and 15.8% m/m is AT.

[c]βCD-AT-AuNPs mass; considering 0.7% m/m is Au, which have been calculated using atomic absorption. Therefore, 99.3% m/m corresponds to βCD-AT complex in the ternary system.

Fig B shows that the functionalization with βCD and AuNPs does not affect the antibacterial activity of AT (MIC = 64 μg/ml). This indicates that the compound can be released from the system and exert its activity against *Enterococcus faecalis*.

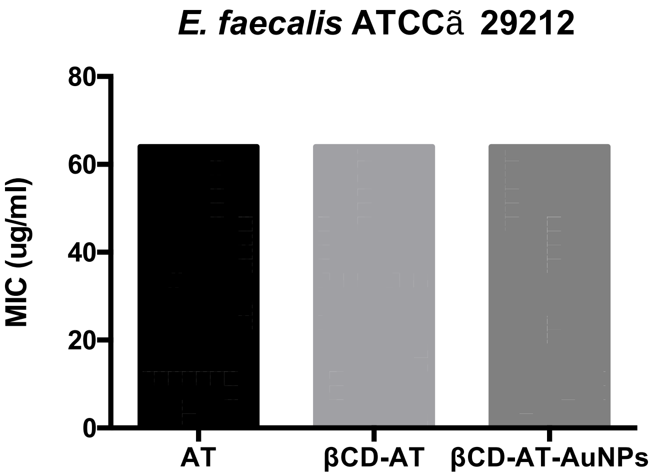

Fig A. Antibacterial Activity of AT, βCD-AT and βCD-AT-AuNPs. MIC values obtained against *Enterococcus faecalis*.
